# Bad Ragaz Ring Method Versus Halliwick Method in Verbally Cooperative Children with Spastic Cerebral Palsy (GMFCS Levels I–II): A Pilot Comparison Study

**DOI:** 10.3390/jcm15176632

**Published:** 2026-08-27

**Authors:** Na-Yeon Ye, Eun-Ju Lee

**Affiliations:** 1 Department of Physical Therapy, Graduate School of Life and Health, Kyungsung University, Busan 48434, Republic of Korea; yny1997@naver.com; 2 Department of Physical Therapy, Kyungsung University, Busan 48434, Republic of Korea

**Keywords:** Bad Ragaz Ring Method, Halliwick method, cerebral palsy, aquatic therapy, proprioceptive neuromuscular facilitation, muscle thickness, balance, pediatric rehabilitation, pilot study

## Abstract

**Background/Objectives**: Aquatic therapy is widely used for children with cerebral palsy (CP), but existing approaches such as the Halliwick method focus primarily on postural control and mobility with limited active resistance exercise. The Bad Ragaz Ring Method (BRRM) integrates proprioceptive neuromuscular facilitation (PNF) diagonal patterns with closed kinetic chain (CKC) exercise in the aquatic environment, yet its direct application to children with spastic CP has rarely been examined. This pilot study compared the effects of BRRM and the Halliwick method on muscle thickness, strength, and balance in verbally cooperative children with spastic CP. **Methods**: Twelve children with spastic CP (Gross Motor Function Classification System [GMFCS] Levels I–II, aged 5–18 years) were randomly allocated to the BRRM group (*n* = 6) or the Halliwick group (*n* = 6), stratified by GMFCS level. Both groups received 40-min sessions twice weekly for 8 weeks (16 sessions). Transversus abdominis (TrA) and tibialis anterior (TA) muscle thickness, ankle dorsiflexion (ADF) strength, one-leg stance (OLS) duration, and Timed Up and Go (TUG) performance were assessed at baseline and after 2, 4, 6, and 8 weeks. A linear mixed model was used to analyze group × time interactions. **Results**: Significant group × time interactions were observed for TrA thickness (*p* = 0.02, partial *η*^2^ = 0.65), ADF strength (*p* < 0.01, partial *η*^2^ = 0.95), and OLS duration (*p* = 0.02, partial *η*^2^ = 0.66), with larger changes in the BRRM group. No significant between-group differences were found for TA thickness or TUG performance. **Conclusions**: In verbally cooperative children with spastic CP at GMFCS Levels I–II, BRRM was associated with larger changes than the Halliwick method in deep trunk muscle thickness, ankle dorsiflexion strength, and static balance. These preliminary findings support further investigation of BRRM in larger randomized controlled trials.

## 1. Introduction

Cerebral palsy (CP) is a neurodevelopmental disorder resulting from a non-progressive lesion to the immature brain, characterized by permanent disturbances of movement and posture, often accompanied by disturbances of cognition, communication, sensation, perception, and behavior [1,2]. Among the recognized motor subtypes (spastic, dyskinetic, ataxic, and mixed), the spastic type is the most prevalent, accounting for approximately 70–80% of all cases [3].

In spastic CP, damage to the upper motor neuron pathway produces abnormal hyperexcitability of alpha motor neurons, resulting in increased muscle tone [4]. This is clinically manifested as muscle spasms, exaggerated reflex responses, and impaired intermuscular coordination [5]. In particular, abnormal co-contraction of agonist and antagonist muscles disrupts selective motor control [6], leading to reduced balance capacity, muscle weakness, atrophy, and soft-tissue contracture. Furthermore, limited early spontaneous movement restricts sensorimotor experience, impairing the development of head–trunk alignment control, proximal co-contraction, and body awareness, which in turn reduces core stability [7]. Consequently, the thickness and activation of deep trunk stabilizing muscles, most notably the transversus abdominis (TrA), are diminished, negatively affecting postural stability and body alignment.

These neurological impairments manifest as biomechanical imbalances at both the proximal trunk and distal limb segments. At the trunk, chronic crouched sitting posture is associated with a reduction in TrA thickness [8,9]. Distally, weakness of the tibialis anterior (TA), secondary to antagonistic spasticity of the plantarflexors (gastrocnemius and soleus), produces foot drop during swing phase and reduced shock absorption during stance, culminating in a toe-walking gait pattern [10]. These structural deficits and abnormal motor control patterns compromise postural control and balance [11]. TrA thickness, TA thickness, and ankle dorsiflexion strength therefore serve as clinical indicators of trunk stability and gait pattern in children with spastic CP. Interventions that combine deep trunk muscle activation with distal strengthening have been proposed to address both components.

Aquatic therapy offers therapeutic advantages through three physical properties: buoyancy, viscosity, and hydrostatic pressure. Buoyancy reduces joint loading, facilitating muscle relaxation, increased range of motion, and reduced tone in antigravity muscles [12,13]; viscosity provides omnidirectional, inelastic resistance proportional to movement velocity, enabling safe strengthening stimuli for weakened muscles; and hydrostatic pressure provides uniform trunk compression that enhances postural stability and proprioceptive input [14,15]. The Halliwick method, a ten-point program progressing from mental adjustment to independent locomotion, is among the most widely applied aquatic techniques in pediatric CP rehabilitation, utilizing buoyancy, wave, turbulence, and rotational responses to promote postural control and motor learning [16,17]. Its emphasis is on postural control and mobility, with comparatively limited application of active resistance exercise.

The Bad Ragaz Ring Method (BRRM) incorporates active resistance in the aquatic environment. BRRM involves a therapist providing manual resistance while the patient is supported in a supine floating position using ring flotation devices, integrating diagonal and spiral patterns of proprioceptive neuromuscular facilitation (PNF) with closed kinetic chain (CKC) exercise [18]. Irradiation and reciprocal inhibition have been proposed as mechanisms by which BRRM may activate weakened distal muscles such as the TA while reducing antagonist spasticity, and aquatic CKC application may enhance proprioception and joint stability through activation of deep trunk stabilizers such as the TrA [19]. The aquatic environment may also support the application of PNF patterns, as buoyancy reduces antigravity demand and viscosity provides accommodating, velocity-dependent resistance [20].

BRRM imposes relatively high cognitive demands, requiring patients to comprehend therapist instructions and actively perform resisted movement patterns [18]. Because children with CP frequently present with concomitant cognitive limitations [21], prior BRRM research has been largely confined to adult populations with stroke or musculoskeletal conditions [22,23,24]. Aquatic intervention studies in children with CP have predominantly examined general aquatic exercise or the Halliwick method, and studies examining outcomes ranging from structural indicators, such as TrA and TA thickness, to balance performance are lacking [25]. Among children with spastic CP, those with relatively preserved cognitive function who can comprehend therapist instructions and perform active resistance exercise may be able to participate in BRRM. The pilot study compared the effects of BRRM and the Halliwick method in independently ambulatory children with spastic CP at Gross Motor Function Classification System (GMFCS) Levels I–II who were capable of following motor instructions. The outcomes examined were the thickness of a core stabilizing muscle (transversus abdominis) and a distal muscle (tibialis anterior), ankle dorsiflexion strength, and static and dynamic balance.

## 2. Materials and Methods

### 2.1. Participants

Twelve children aged 5–18 years with spastic cerebral palsy (CP) who met the eligibility criteria were recruited from patients attending Center B, an aquatic therapy center located in city D, Republic of Korea. General characteristics, comprising sex, age, height, weight, and site of paralysis, were collected through guardian interviews. Motor function was classified using the Gross Motor Function Classification System (GMFCS). Baseline characteristics and Special Education Screening Test (SEST) scores are presented in Table 1.

**Figure 1:**
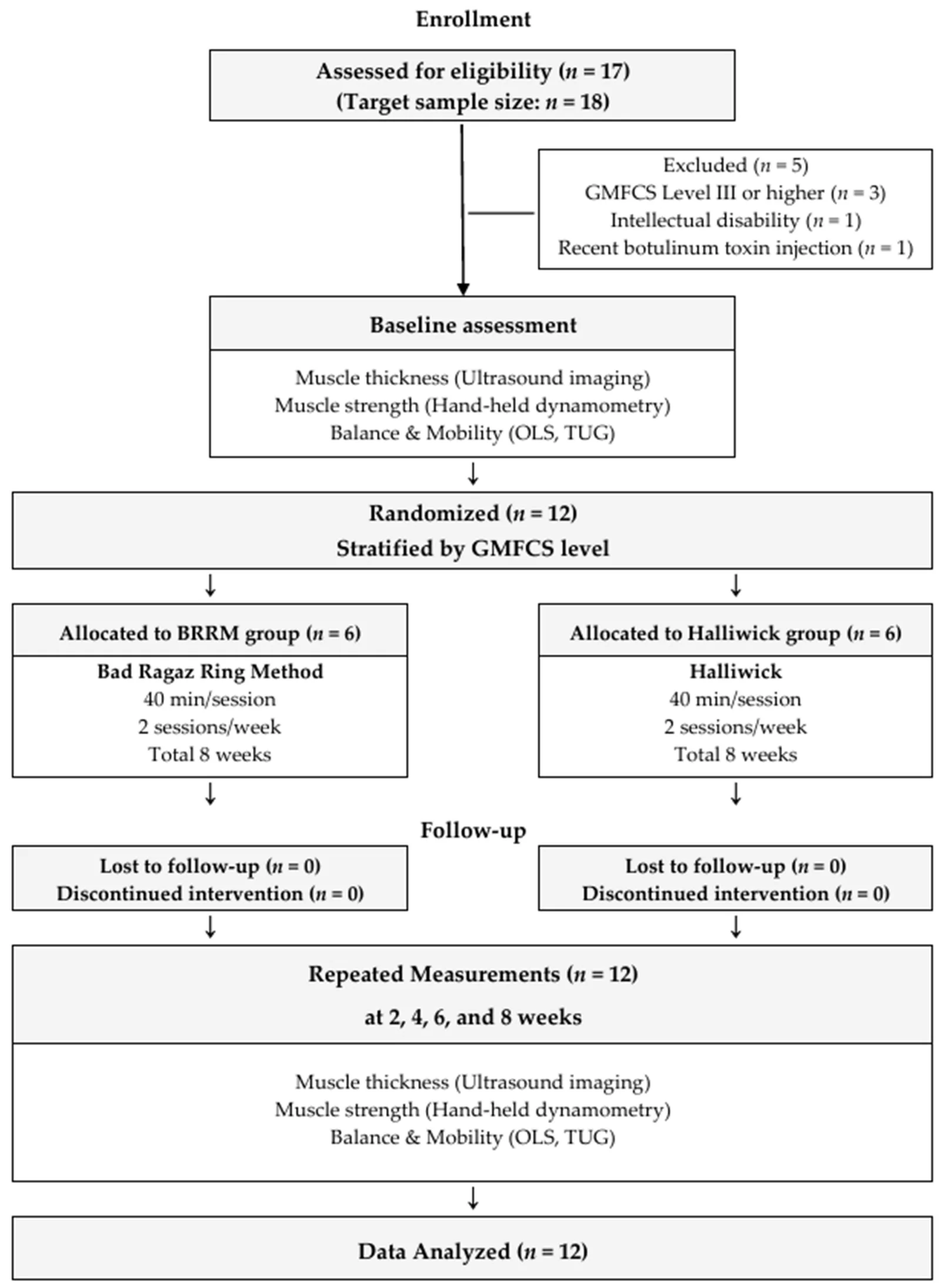
CONSORT flow diagram of participant recruitment, allocation, and analysis. GMFCS, Gross Motor Function Classification System; BRRM, Bad Ragaz Ring Method; OLS, one-leg stance; TUG, Timed Up and Go.

An a priori sample size calculation was performed using G*Power 3.1.9.7 (paired *t*-test, α = 0.05, power = 0.80), assuming a large effect size (Cohen’s *d* = 0.8). This indicated a minimum of 15 participants; allowing for 20% attrition, the protocol approved by the Institutional Review Board planned to enroll 18 children. During the recruitment period, 17 children attending the center were screened, of whom 5 did not meet the eligibility criteria. Twelve children were enrolled and randomized, none withdrew, and data from all 12 were analyzed (Figure 1). Although this fell short of the planned enrollment, the resulting group size was comparable to that of a previous study of aquatic exercise therapy in children with CP [26]. The primary analysis reported here is the group × time interaction, rather than the within-group comparison for which the sample size was calculated, and the study was not formally powered for this analysis; the trial is therefore reported as a pilot study in accordance with the CONSORT extension for randomized pilot and feasibility trials [27]. Prior to enrollment, the study purpose, procedures, the nature of the aquatic interventions, and the right to withdraw at any time were explained to participants and their legal guardians, and voluntary written informed consent was obtained.

Inclusion criteria were: (1) diagnosis of spastic CP by a physiatrist; (2) GMFCS Level I or II, indicating independent standing and walking ability with or without an assistive device; (3) lower-limb muscle tone of Grade 2 or below on the Modified Ashworth Scale (MAS), sufficient to perform aquatic resistance exercise; and (4) scores below 4 points in the memory/attention/perception (MAP), cognitive, and communication domains of the SEST developed by the Korean National Institute of Special Education [28]. On this test, higher scores indicate a greater likelihood of requiring further diagnostic evaluation; scores below 4 points were therefore taken to indicate sufficient capacity to follow active motor instructions. Exclusion criteria were: (1) severe cognitive or behavioral impairment (e.g., intellectual disability, autism spectrum disorder) precluding active participation in therapy; (2) orthopedic lower-limb surgery or botulinum toxin injection within the preceding 6 months, which could induce transient changes in muscle status; and (3) inability to control bowel or bladder function, precluding adequate infection control and safety in the aquatic environment.

### 2.2. Study Design

A randomized comparison design with repeated measures was used to compare the effects of the Bad Ragaz Ring Method (BRRM) and the Halliwick method. All 12 eligible participants were enrolled and assessed at baseline before allocation. They were then separated into two strata by GMFCS level, eight children at Level I and four at Level II, and within each stratum each child drew a lot that determined the group assignment. No randomization software was used, no block size was specified, and allocation was not concealed by sealed envelopes or a computer-generated sequence. This produced a BRRM group (*n* = 6) and a Halliwick group (*n* = 6), each comprising 4 children at Level I and 2 at Level II. Stratification by GMFCS level was used to balance functional levels across the two groups. Both groups received aquatic intervention twice weekly for 40 min per session over 8 weeks (16 sessions total). Assessments were repeated at five time points: baseline and after 2, 4, 6, and 8 weeks of intervention (Figure 1). The therapist who delivered the interventions also performed all assessments. Neither the therapist nor the participants were blinded to group allocation, and ultrasound images were analyzed without masking of group allocation.

### 2.3. Aquatic Interventions

All aquatic interventions were conducted in the aquatic rehabilitation pool (6 m × 4 m, 120 cm depth) at Center B, with water temperature maintained at 32–33 °C. Each 40-min session comprised a 5-min warm-up and 5-min cool-down applied identically to both groups, and a 30-min core intervention consisting of either BRRM or the Halliwick method. All sessions were delivered by a single International Aquatic Therapy Faculty certified physical therapist with over 7 years of clinical experience in pediatric aquatic rehabilitation.

#### 2.3.1. Warm-Up and Cool-Down

Warm-up consisted of therapist-led passive leg stretching in a ring-supported supine float, to prevent injury and relax the legs before the main exercise. Cool-down consisted of guided diaphragmatic breathing in the same floating position, to relieve muscle tension and promote relaxation.

#### 2.3.2. Main Aquatic Exercise

BRRM group. Three PNF-based patterns were applied using ring flotation and manual resistance: ① Trunk lateral flexion pattern, targeting deep trunk stabilizers (e.g., transversus abdominis)—the child, floated supine with rings at the neck, pelvis, and ankles, curled the trunk to bring one shoulder toward the opposite hip against the therapist’s manual resistance, producing combined trunk flexion, lateral flexion, and rotation. ② Bilateral symmetrical leg pattern, targeting leg strength and selective activation of the ankle dorsiflexors (e.g., tibialis anterior) with trunk co-contraction—from hip extension–adduction–external rotation, the child flexed both legs together against water and manual resistance into hip flexion–abduction–internal rotation with ankle dorsiflexion. ③ Bilateral reciprocal leg pattern, targeting pelvic stability and dynamic balance via gait-like movement—the therapist guided one leg into hip/knee flexion while providing stabilizing resistance to the extended contralateral leg, alternating in a reciprocal pattern.

Halliwick group. Three sequential stages were applied: ① Mental adjustment—breath-control drills (e.g., blowing bubbles) and active kicking to reduce fear of water and build familiarity with buoyancy and resistance. ② Trunk control—from a prone float supported at the therapist’s shoulders, the child flexed the hips and trunk, pushed off the therapist, and progressed to independent standing. ③ Balance control—forward walking and repeated forward jumping against water resistance and perturbation, with minimal or no therapist assistance, to improve dynamic balance and functional mobility.

### 2.4. Outcome Measures

#### 2.4.1. Muscle Thickness Measurement

Muscle thickness was measured at each of the five assessment time points using an ultrasound imaging device (SONON-300L, Healcerion, Seoul, Republic of Korea). Images were acquired in dual mode using a 10 MHz linear probe, and thickness analysis of the captured images was performed using ImageJ software, version 1.54 (National Institutes of Health, Bethesda, MD, USA). Sufficient ultrasound gel was applied to minimize mechanical compression of the skin by the probe, and the probe was maintained perpendicular to the skin surface to prevent image distortion. All measurements were obtained at rest, without muscle contraction, and the mean of three repeated measurements at each site was used for analysis.

Transversus abdominis (TrA): Participants assumed a hook-lying position, and images were captured at the end of a comfortable expiration to obtain a consistent abdominal muscle thickness. The probe was placed transversely at a point approximately 2.5 cm anteromedial to the midpoint of the line connecting the 12th rib and the iliac crest. Tibialis anterior (TA): Participants assumed a supine position with a towel placed under the knee to maintain a comfortable, slightly flexed position. The probe was placed longitudinally along the anterior tibial crest at the point corresponding to 20% of the total distance from the fibular head to the lateral malleolus, measured from the proximal end.

#### 2.4.2. Muscle Strength Measurement

Lower-limb muscle strength was measured in newtons (N) using a hand-held dynamometer (Power Track II Commander, JTECH Medical, Midvale, UT, USA). This device has been reported to show intra-rater reliability of 0.73–0.95 and inter-rater reliability of 0.62–0.96 [29,30]. Ankle dorsiflexion strength was measured with the child in a supine position, the knee flexed to approximately 30°, and the ankle starting from a neutral position. The sensor pad was placed on the dorsum of the foot (metatarsal region), and the child was verbally instructed to “pull your ankle up as hard as you can.” Strength was measured using the “make test” method, in which the child performed a maximal isometric contraction against the examiner’s resistance for approximately 5 s. A 30-s rest was provided between trials to prevent error from muscle fatigue, and the mean of three repeated measurements was used for analysis.

#### 2.4.3. Balance Measurement

One-leg stance test (OLS): Static balance was assessed using the OLS. Participants stood on a flat, firm surface with the supporting knee fully extended and the contralateral hip and knee each flexed to 90°, arms relaxed at the sides, and gaze directed forward. The duration from assumption of the position until loss of balance or contact of the raised foot with the ground was recorded, and the mean of three trials was used for analysis. Timed Up and Go test (TUG): Dynamic balance was assessed using the TUG. Participants sat in a chair with a backrest, hands resting lightly on the thighs, and upon the examiner’s “start” command, rose from the chair, walked forward to a marker 3 m away, turned, returned to the chair, and sat down completely. The total time was recorded, and the mean of three trials was used for analysis. This test has been reported to show excellent intra-rater (*r* = 0.99) and inter-rater (*r* = 0.98) reliability [31].

### 2.5. Statistical Analysis

All data were analyzed using SPSS Statistics version 26.0 (IBM Corp., Armonk, NY, USA). General characteristics were summarized using descriptive statistics, with continuous variables presented as mean ± standard deviation and categorical variables as frequencies and percentages. Baseline characteristics were compared between groups; given the small sample size (*n* = 6 per group), the Mann-Whitney *U* test was used for continuous variables and Fisher’s exact test for categorical variables.

Intra-rater reliability was assessed for all outcome measures using the three consecutive measurements obtained at baseline. Intraclass correlation coefficients were calculated from a two-way mixed-effects model with absolute agreement. Both single-measure (ICC_3,1_) and average-measure (ICC_3,3_) coefficients are reported; because the mean of three measurements was used in the analysis, ICC_3,3_ was taken as the primary reliability index. The standard error of measurement (SEM) was calculated as SD × √(1 − ICC), and the minimal detectable change at the 95% confidence level (MDC_95_) as 1.96 × √2 × SEM (Table 2).

A linear mixed model (LMM) was applied to examine changes over time and intervention effects. Group, time, and the group × time interaction were entered as fixed effects, and participant was entered as a random effect to account for individual differences in baseline values and response variability. Interpretation of intervention effects was based on the group × time interaction (Table 3). Estimated marginal means with 95% confidence intervals were derived from the model for each group at each time point (Table 4). To evaluate the magnitude of effects beyond statistical significance, partial eta-squared (partial *η*^2^) was calculated for fixed effects using the F-statistic and degrees of freedom from the LMM, and interpreted according to the criteria of Richardson [32]: 0.01 as a small effect, 0.06 as a medium effect, and 0.14 or greater as a large effect. Change scores and percentage change from baseline to 8 weeks were calculated for each group. Statistical significance was set at *p* < 0.05 for all analyses.

## 3. Results

### 3.1. General Characteristics of Participants and Baseline Homogeneity

A total of 12 children with spastic cerebral palsy were enrolled, with 6 allocated to the BRRM group and 6 to the Halliwick group. Mean age was 12.83 ± 3.60 years in the BRRM group and 12.00 ± 4.29 years in the Halliwick group; height was 144.17 ± 17.51 cm and 139.00 ± 19.31 cm, and weight was 41.50 ± 15.37 kg and 39.67 ± 12.71 kg, respectively. GMFCS level (I/II) was identical between the groups by design, as randomization was stratified by GMFCS. Type of paralysis (diplegia/hemiplegia) and lower-limb muscle tone (Modified Ashworth Scale Grade 1/1+) also showed identical distributions. Pre-intervention SEST scores were compared across the MAP, cognitive, and communication domains. No significant between-group differences were found for any general characteristic or cognitive screening domain (all *p* > 0.05; Table 1).

### 3.2. Intra-Rater Reliability of Outcome Measures

Intra-rater reliability, standard error of measurement, and minimal detectable change for all outcome measures are presented in Table 2. ICC_3,3_ values for the mean of three measurements ranged from 0.906 (TrA) to 0.990 (OLS). The 8-week changes observed in both groups exceeded the corresponding MDC_95_ values for all outcomes.

### 3.3. Changes in Muscle Thickness

**Figure 2:**
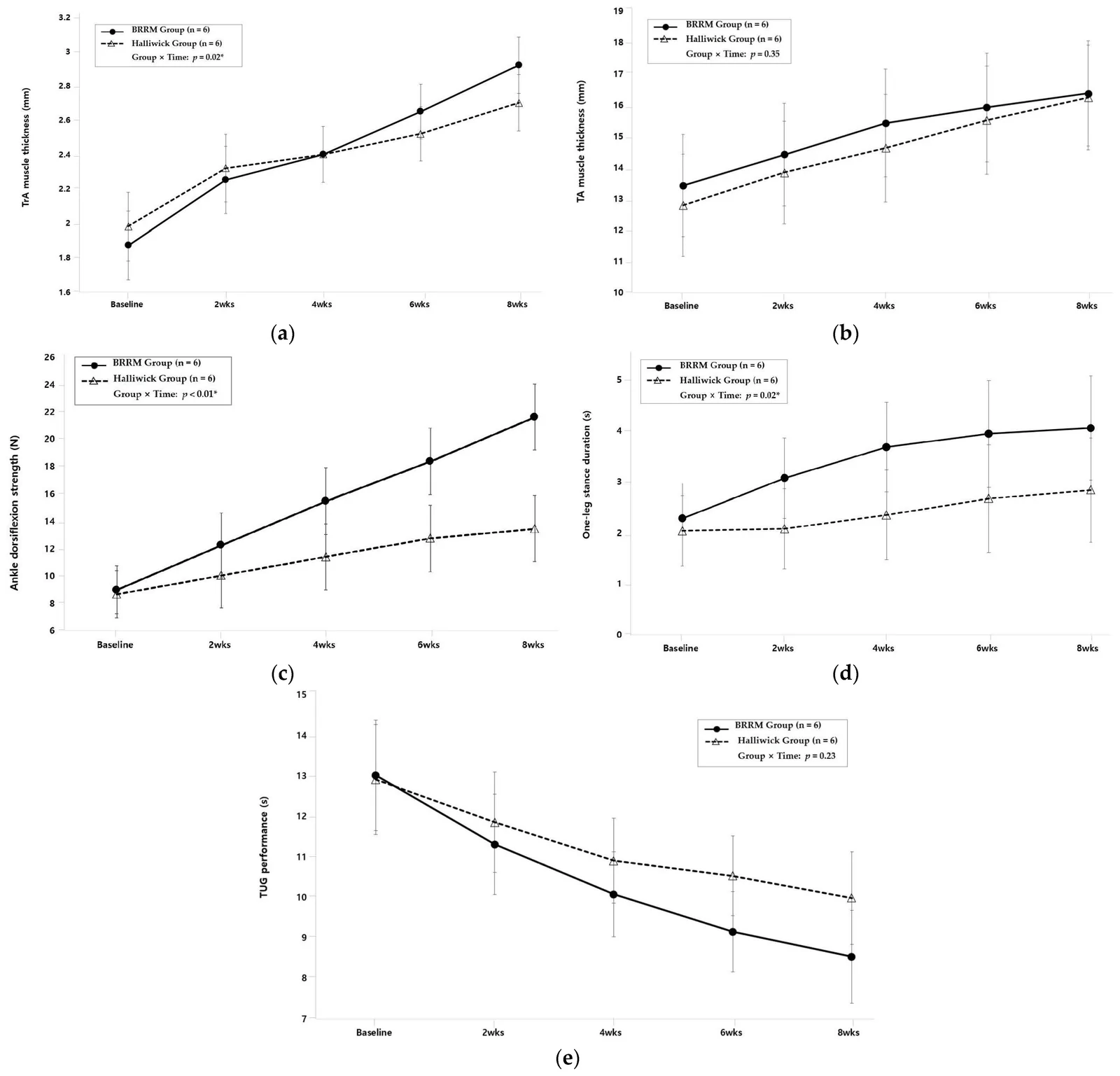
Changes in outcome measures over 8 weeks between the BRRM and Halliwick groups: (**a**) transversus abdominis (TrA) muscle thickness (mm); (**b**) tibialis anterior (TA) muscle thickness (mm); (**c**) ankle dorsiflexion strength (N); (**d**) one-leg stance duration (s); (**e**) Timed Up and Go (TUG) performance (s). Values are estimated marginal means with 95% confidence intervals derived from a linear mixed model. The number of participants per group is shown in the legend; no participant withdrew, and all 12 were analyzed at every time point. For TUG, lower values indicate better performance. * Significant group × time interaction (*p* < 0.05). BRRM, Bad Ragaz Ring Method.

Transversus abdominis (TrA) thickness. Both groups showed a continuous increase in TrA thickness over the intervention period (Figure 2a). The change from baseline to 8 weeks was +1.05 mm (56.24%) in the BRRM group and +0.72 mm (36.16%) in the Halliwick group, a difference of 20.08 percentage points (Table 3). The linear mixed model (LMM) analysis showed no significant main effect of group (*p* = 0.68, partial *η*^2^ = 0.02) and a significant main effect of time (*p* < 0.01, partial *η*^2^ = 0.97). The group × time interaction was significant with a large effect size (*p* = 0.02, partial *η*^2^ = 0.65).

Tibialis anterior (TA) thickness. Both groups showed a gradual increase in TA thickness over the intervention period (Figure 2b). The change from baseline to 8 weeks was +2.95 mm (21.96%) in the BRRM group and +3.45 mm (26.95%) in the Halliwick group (Table 3). The LMM analysis showed no significant main effect of group (*p* = 0.63, partial *η*^2^ = 0.02) and a significant main effect of time (*p* < 0.01, partial *η*^2^ = 0.98). The group × time interaction was not significant (*p* = 0.35, partial *η*^2^ = 0.33).

### 3.4. Changes in Muscle Strength

Ankle dorsiflexion (ADF) strength. Both groups showed an increase in ADF strength over the intervention period, with a steeper increase in the BRRM group (Figure 2c). The change from baseline to 8 weeks was +12.62 N (140.45%) in the BRRM group and +4.80 N (55.38%) in the Halliwick group, a difference of 85.07 percentage points (Table 3). The LMM analysis showed a significant main effect of group (*p* < 0.01, partial *η*^2^ = 0.49) and a significant main effect of time (*p* < 0.01, partial *η*^2^ = 0.99). The group × time interaction was significant with a large effect size (*p* < 0.01, partial *η*^2^ = 0.95).

### 3.5. Changes in Balance

One-leg stance (OLS, static balance). Both groups showed an increase in OLS duration over the intervention period, with a greater increase in the BRRM group (Figure 2d). The change from baseline to 8 weeks was +1.77 s (77.70%) in the BRRM group and +0.80 s (39.11%) in the Halliwick group, a difference of 38.59 percentage points (Table 3). The LMM analysis showed no significant main effect of group (*p* = 0.09, partial *η*^2^ = 0.26) and a significant main effect of time (*p* < 0.01, partial *η*^2^ = 0.82). The group × time interaction was significant with a large effect size (*p* = 0.02, partial *η*^2^ = 0.66).

Timed Up and Go (TUG, dynamic balance). Both groups showed a continuous reduction in TUG performance time over the intervention period (Figure 2e). The change from baseline to 8 weeks was −4.47 s (−34.37%) in the BRRM group and −2.93 s (−22.71%) in the Halliwick group (Table 3). The LMM analysis showed no significant main effect of group (*p* = 0.25, partial *η*^2^ = 0.13) and a significant main effect of time (*p* < 0.01, partial *η*^2^ = 0.94). The group × time interaction was not significant (*p* = 0.23, partial *η*^2^ = 0.40).

Estimated marginal means with 95% confidence intervals at each time point are presented in Table 4.

## 4. Discussion

This study examined the effects of the Bad Ragaz Ring Method (BRRM) on muscle thickness, strength, and balance in children with spastic cerebral palsy (CP) capable of following motor instructions, in comparison with the Halliwick method. Given that prior aquatic therapy research in pediatric CP has largely focused on general aquatic exercise or the Halliwick method [33], the present study applied BRRM, which integrates proprioceptive neuromuscular facilitation (PNF) diagonal patterns and closed kinetic chain (CKC) exercise, to this population and evaluated both structural and functional outcomes. Significant group × time interactions were observed for transversus abdominis (TrA) thickness, ankle dorsiflexion (ADF) strength, and one-leg stance (OLS) duration, with larger changes in the BRRM group. For tibialis anterior (TA) thickness and Timed Up and Go (TUG) performance, both groups improved over time, but the group × time interaction was not significant.

The between-group differences observed in three of the five outcomes may be related to the distinct characteristics of the two interventions. Whereas the Halliwick method emphasizes postural control and mobility, BRRM combines the diagonal and spiral patterns of PNF with CKC exercise in water. Several mechanisms described in previous literature may account for this difference, although none were evaluated in the present study. Water resistance increases proportionally with movement velocity, and the turbulence generated during aquatic movement may impose repeated demands on trunk stabilizing muscles [34]. When the distal segment is relatively fixed by the therapist’s contact point while the body moves, proximal trunk stability may be recruited anticipatorily, inducing co-contraction of the deep proximal muscles [35]. Resistive stimuli applied to one muscle group may also spread neuromuscular excitation to adjacent muscle groups, a phenomenon described as irradiation [36]. These are hypothetical explanations rather than mechanisms measured in this study.

The greater increase in TrA thickness in the BRRM group may be related to the mechanisms described above. TrA is a deep stabilizing muscle that is anticipatorily activated prior to limb movement to secure segmental stability of the lumbopelvic complex [37,38]. One possible explanation is that maintaining trunk stability against aquatic turbulence and resistance in a CKC context involves repeated co-contraction of the TrA, which may contribute to increased muscle thickness. These observations are consistent with previous reports of increased trunk strength and endurance after BRRM [23,39] and increased trunk muscle co-contraction in the aquatic environment during BRRM [40]. Outside the aquatic setting, CKC exercise has been reported to induce co-contraction of deep stabilizers such as the TrA and multifidus [41].

The increase in ADF strength in the BRRM group (change rate 140.45% vs. 55.38%) may be explained by PNF diagonal patterns recruiting multiple muscle groups simultaneously, which has been associated with increased motor unit recruitment and neuromuscular coordination [42,43]. One possible mechanism is reciprocal innervation, whereby contraction of the agonist (tibialis anterior) is accompanied by inhibition of the antagonist (triceps surae) [36]. CKC exercises have also been reported to promote cooperative contraction between agonists and antagonists, enhancing joint stability and increasing lower-limb muscle activity [22,44,45,46,47]. Neither motor unit recruitment nor reciprocal inhibition was measured in the present study.

This between-group difference in ADF strength was not accompanied by a corresponding difference in TA thickness, which increased in both groups without a significant group × time interaction. One possible explanation is that the strength gains reflected neural rather than structural adaptation. Strength gains during the initial weeks of resistance training have been attributed primarily to neurological adaptations, including increased motor unit recruitment, firing rate, and agonist–antagonist coordination, with hypertrophy emerging over longer periods [48,49,50]. Previous studies reporting increased muscle volume in children with spastic CP employed progressive overload targeting hypertrophy over intervention periods of 12 weeks or longer [51,52], whereas the BRRM protocol in this study emphasized trunk stability, balance, and neuromuscular control rather than selective high-intensity resistance to the TA. The skeletal muscles of children with CP have been described as showing structural degeneration, including reduced muscle mass, thickness, and fiber length, together with increased fatty and connective tissue infiltration [53,54]. In the present study, TA thickness increased in both groups over the 8-week period (+2.95 mm and +3.45 mm), exceeding the MDC_95_ obtained in this study, although the group × time interaction was not significant. As both groups received an aquatic intervention and no non-exercising comparison group was included, the contribution of growth or maturation to this increase cannot be determined.

The greater increase in OLS duration in the BRRM group may be related to the changes observed in trunk muscle thickness. The OLS task, in which the base of support is restricted to a single leg, requires trunk alignment, pelvic control, and coordinated contraction of the lower-limb muscles to maintain the center of mass [55,56]. Insufficient trunk stability has been associated with increased postural sway and shortened balance duration [57]. One possible interpretation is that the increase in TrA thickness in the BRRM group accompanied improved trunk stabilization, consistent with the kinetic chain principle that proximal stability underlies distal functional performance; however, this relationship was not tested directly in the present study. In addition, the rhythmic stabilization technique of PNF, which has been reported to improve postural control through repetitive alternating isometric contractions [36], is incorporated in the BRRM procedure in which the therapist applies resistance from various directions. This is consistent with a case report in which PNF training improved balance and gait in a child with spastic CP [58].

By contrast, TUG performance time improved in both groups without a between-group difference. The TUG is a composite task involving sit-to-stand, walking, turning, and sitting, and measures dynamic balance and functional mobility [59]. Unlike the static OLS task, TUG depends more heavily on gait speed and postural control during ambulation. Both groups may have derived similar benefits from the aquatic environment through buoyancy-induced reduction in weight-bearing and viscosity-induced safe movement conditions. Given the repetitive and automated nature of gait, the difference in trunk muscle activation between the two interventions may not have been sufficient to produce a between-group difference in gait efficiency within this intervention period. This pattern, significant improvement in static but not dynamic balance, may reflect task specificity, whereby intervention effects manifest according to the characteristics of the trained task.

Established minimal clinically important difference (MCID) values for TrA thickness, ADF strength, or OLS duration in children with CP were not identified, and the clinical importance of the observed changes in these outcomes therefore cannot be assessed against published benchmarks. For the TUG, MDC_95_ values of 1.40 s and 2.87 s and anchor-based MCID estimates of 1.12 s and 2.68 s have been reported for children with CP at GMFCS Levels I and II, respectively [60]. Using a distribution-based method, MCID estimates of 1.1 s to 1.7 s for Level I and 0.7 s to 1.2 s for Level II have also been reported for a modified version of the TUG in children aged 8 to 18 years with bilateral spastic CP [61]. The reductions observed in the present study (−4.47 s in the BRRM group and −2.93 s in the Halliwick group) exceeded all of these values, although the change in the Halliwick group only slightly exceeded the MDC_95_ reported for GMFCS Level II [60]. Reported estimates vary with the derivation method, the test version, and the age range of the reference sample.

All observed changes also exceeded the corresponding MDC_95_ values obtained in this study (Table 2). MDC indicates the smallest change that can be distinguished from measurement error and does not by itself establish clinical importance. Whether these changes translate into meaningful gains in daily activities remains to be determined.

These outcome-specific findings may inform the selection of aquatic interventions according to therapeutic goals. BRRM may be considered for children with preserved cognitive function and instruction-following ability whose therapeutic goals include trunk stability, distal muscle strength, and static balance.

This study has several limitations. First, the paucity of prior studies directly applying BRRM to children with spastic CP required indirect interpretation based on PNF and CKC literature, limiting the ability to isolate the unique effects of BRRM itself. Second, the small sample size (*n* = 6 per group) limited statistical power, and the findings should be interpreted as preliminary. Third, no blinding was implemented at any stage. The same therapist delivered both interventions and performed all outcome assessments, and ultrasound images were analyzed without masking of group allocation. Performance bias may have arisen if the therapist’s expectations influenced the delivery of the two interventions, and detection bias may have arisen in outcome measurement and image analysis, particularly for measures involving examiner judgment such as ultrasound thickness and timed balance tasks. Allocation was also not concealed. Fourth, participants were restricted to children at GMFCS Levels I–II with mild spasticity, preserved cognition, and verbal cooperation, and the findings therefore cannot be generalized to children with more severe motor or cognitive impairment. Future research should include multicenter studies with adequate sample sizes, separate the interventionist and the assessor or apply blinded assessment, include participants across a wider range of functional levels, and employ longer intervention periods with post-intervention follow-up to examine the durability of effects and whether improvements in physical function transfer to activities of daily living.

## 5. Conclusions

In children with spastic CP at GMFCS Levels I–II with mild spasticity, preserved cognition, and verbal cooperation, significant group × time interactions were observed for deep trunk muscle thickness, distal muscle strength, and static balance, with larger changes in the BRRM group. No between-group differences were observed for distal muscle thickness or dynamic balance. As a pilot study with a small sample, the findings provide preliminary evidence supporting further investigation of BRRM in larger randomized controlled trials. These findings should not be generalized to children with more severe motor or cognitive impairment.

## Figures and Tables

**Table 1:** General characteristics and baseline homogeneity (*n* = 12).

Variable		BRRM Group *n* (%)	Halliwick Group *n* (%)	Z	*p*
Age (years)		12.83 ± 3.60	12.00 ± 4.29	0.32	0.82
Height (cm)		144.17 ± 17.51	139.00 ± 19.31	0.48	0.70
Weight (kg)		41.50 ± 15.37	39.67 ± 12.71	0.16	0.82
Gender	Male	3 (50.00)	4 (66.67)	-	1.00
	Female	3 (50.00)	2 (33.33)	-	
Type	Diplegia	4 (66.67)	4 (66.67)	-	1.00
	Hemiplegia	2 (33.33)	2 (33.33)	-	
GMFCS	Level I	4 (66.67)	4 (66.67)	-	1.00
	Level II	2 (33.33)	2 (33.33)	-	
MAS	Grade 1	3 (50.00)	3 (50.00)	-	1.00
	Grade 1+	3 (50.00)	3 (50.00)	-	
SEST	MAP	1.17 ± 0.41	1.33 ± 0.52	−0.64	0.59
	Cognitive	0.50 ± 0.55	0.67 ± 0.52	−0.48	0.70
	Communication	0.50 ± 0.84	0.83 ± 0.41	−0.80	0.50

Note. Continuous variables are presented as mean ± standard deviation and categorical variables as *n* (%). Continuous variables were compared using the Mann–Whitney *U* test and categorical variables using Fisher’s exact test. Abbreviations: BRRM, Bad Ragaz Ring Method; GMFCS, Gross Motor Function Classification System; MAS, Modified Ashworth Scale; SEST, Special Education Screening Test; MAP, memory/attention/perception.

**Table 2:** Intra-rater reliability of outcome measures (*n* = 12).

Variable	ICC_3,1_ (95% CI)	ICC_3,3_ (95% CI)	F	SEM	MDC_95_
TrA (mm)	0.763 (0.509–0.916)	0.906 (0.757–0.971)	10.51	0.07	0.20
TA (mm)	0.903 (0.763–0.969)	0.965 (0.906–0.989)	33.96	0.33	0.91
ADF (N)	0.917 (0.614–0.978)	0.971 (0.827–0.993)	82.40	0.50	1.38
OLS (s)	0.971 (0.897–0.992)	0.990 (0.963–0.997)	162.38	0.09	0.25
TUG (s)	0.924 (0.818–0.975)	0.973 (0.931–0.992)	37.45	0.24	0.67

Note. Intraclass correlation coefficients were calculated from a two-way mixed-effects model with absolute agreement, based on three consecutive measurements obtained at baseline. All F tests were significant at *p* < 0.001 with df = 11, 22. SEM and MDC_95_ were calculated from ICC_3,3_, corresponding to the mean of three measurements used in the analysis. Abbreviations: ICC, intraclass correlation coefficient; CI, confidence interval; SEM, standard error of measurement; MDC_95_, minimal detectable change at the 95% confidence level; TrA, transversus abdominis; TA, tibialis anterior; ADF, ankle dorsiflexion; OLS, one-leg stance; TUG, Timed Up and Go.

**Table 3:** Linear mixed model analysis of performance in participants (*n* = 12).

Variable	Effect	df_1_, df_2_	F	*p*	Partial *η^2^*	Change	Change Rate (%)
TrA (mm)	Group	1, 10	0.18	0.68	0.02	-	-
	Time	4, 10	87.37	<0.01 *	0.97	-	-
	Group × Time	4, 10	4.61	0.02 *	0.65	BRRM +1.05 Halliwick +0.72	BRRM 56.24 Halliwick 36.16
TA (mm)	Group	1, 10	0.24	0.63	0.02	-	-
	Time	4, 10	145.44	<0.01 *	0.98	-	-
	Group × Time	4, 10	1.22	0.35	0.33	BRRM +2.95 Halliwick +3.45	BRRM 21.96 Halliwick 26.95
ADF (N)	Group	1, 10	9.40	<0.01 *	0.49	-	-
	Time	4, 10	166.64	<0.01 *	0.99	-	-
	Group × Time	4, 10	44.57	<0.01 *	0.95	BRRM +12.62 Halliwick +4.80	BRRM 140.45 Halliwick 55.38
OLS (s)	Group	1, 10	3.45	0.09	0.26	-	-
	Time	4, 10	11.28	<0.01 *	0.82	-	-
	Group × Time	4, 10	4.76	0.02 *	0.66	BRRM +1.77 Halliwick +0.80	BRRM 77.70 Halliwick 39.11
TUG (s)	Group	1, 10	1.45	0.25	0.13	-	-
	Time	4, 10	36.21	<0.01 *	0.94	-	-
	Group × Time	4, 10	1.67	0.23	0.40	BRRM −4.47 Halliwick −2.93	BRRM −34.37 Halliwick −22.71

Note. * *p* < 0.05. *p*-values smaller than 0.01 are reported as <0.01. Change = 8 weeks—baseline; Change rate (%) = (Change/baseline) × 100. Change and Change rate values are shown only for the Group × Time interaction, indicating between-group differences in longitudinal change. Negative values for TUG indicate improvement (shorter time). Abbreviations: TrA, transversus abdominis; TA, tibialis anterior; ADF, ankle dorsiflexion; OLS, one-leg stance; TUG, Timed Up and Go; BRRM, Bad Ragaz Ring Method; df_1_, numerator degrees of freedom; df_2_, denominator degrees of freedom; F, F-statistic; Partial *η^2^*, partial eta-squared.

**Table 4:** Estimated marginal means (95% confidence intervals) from the linear mixed model (*n* = 6 per group).

Variable	Group	Baseline	2 Weeks	4 Weeks	6 Weeks	8 Weeks
TrA (mm)	BRRM	1.87 (1.67–2.07)	2.25 (2.05–2.45)	2.40 (2.24–2.56)	2.65 (2.49–2.81)	2.92 (2.75–3.08)
	Halliwick	1.98 (1.78–2.18)	2.32 (2.12–2.51)	2.40 (2.24–2.56)	2.52 (2.36–2.68)	2.70 (2.54–2.87)
TA (mm)	BRRM	13.43 (11.80–15.06)	14.42 (12.78–16.06)	15.43 (13.71–17.16)	15.93 (14.20–17.67)	16.38 (14.70–18.06)
	Halliwick	12.80 (11.17–14.43)	13.85 (12.21–15.49)	14.63 (12.91–16.36)	15.52 (13.78–17.25)	16.25 (14.57–17.93)
ADF (N)	BRRM	8.98 (7.24–10.73)	12.25 (9.88–14.62)	15.47 (13.05–17.88)	18.37 (15.93–20.80)	21.60 (19.19–24.01)
	Halliwick	8.67 (6.92–10.41)	10.05 (7.68–12.42)	11.40 (8.99–13.81)	12.75 (10.31–15.19)	13.47 (11.06–15.88)
OLS (s)	BRRM	2.28 (1.60–2.97)	3.08 (2.29–3.86)	3.69 (2.81–4.57)	3.94 (2.89–4.99)	4.06 (3.03–5.08)
	Halliwick	2.04 (1.36–2.73)	2.09 (1.30–2.87)	2.35 (1.48–3.23)	2.67 (1.62–3.72)	2.84 (1.82–3.86)
TUG (s)	BRRM	13.01 (11.65–14.37)	11.31 (10.08–12.55)	10.08 (9.03–11.13)	9.15 (8.16–10.14)	8.54 (7.40–9.68)
	Halliwick	12.92 (11.56–14.28)	11.85 (10.61–13.09)	10.91 (9.86–11.95)	10.53 (9.54–11.52)	9.98 (8.84–11.13)

Note. Values are estimated marginal means (95% confidence intervals) from a linear mixed model (*n* = 6 per group; no missing data). Confidence intervals are based on the pooled model error (df = 10). Lower TUG values indicate better performance. Fixed-effect tests are presented in Table 3. Abbreviations: TrA, transversus abdominis; TA, tibialis anterior; ADF, ankle dorsiflexion; OLS, one-leg stance; TUG, Timed Up and Go; BRRM, Bad Ragaz Ring Method.

## Data Availability

The data presented in this study are available on request from the corresponding author due to privacy and ethical restrictions related to the pediatric study population.

## Abbreviations

The following abbreviations are used in this manuscript:

ADF	Ankle dorsiflexion
BRRM	Bad Ragaz Ring Method
CI	Confidence interval
CKC	Closed kinetic chain
CP	Cerebral palsy
GMFCS	Gross Motor Function Classification System
IATF	International Aquatic Therapy Faculty
LMM	Linear mixed model
MAP	Memory/attention/perception
MAS	Modified Ashworth Scale
MCID	Minimal clinically important difference
MDC_95_	Minimal detectable change at the 95% confidence level
OLS	One-leg stance
PNF	Proprioceptive neuromuscular facilitation
SEST	Special Education Screening Test
TA	Tibialis anterior
TrA	Transversus abdominis
TUG	Timed Up and Go
